# Modeling molecular environment of allomorphic *N*-salicylidene-4-halo-aniline crystals through iterative QM/QM′ structural optimization

**DOI:** 10.1007/s00894-025-06579-2

**Published:** 2025-11-27

**Authors:** Mingge Xu, Masahiro Suzuki, Ryo Koibuchi, Isao Yoshikawa, Hirohiko Houjou

**Affiliations:** 1 Institute of Industrial Science, The University of Tokyo, 4-6-1 Komaba, Meguro-Ku, Tokyo, 153-8505 Japan; 2 Environmental Science Center, The University of Tokyo, 7-3-1 Hongo, Bunkyo-Ku, Tokyo, 113-0033 Japan

**Keywords:** Crystal engineering, Chromism, Schiff base, Polymorphism, Normal-mode analysis

## Abstract

**Context:**

Two allomorphic crystals, photochromic *N*-salicylidene-4-bromoaniline (α-SA4B) and non-photochromic *N*-salicylidene-4-chloroaniline (β-SA4C), were simulated using a hierarchical hybrid quantum mechanical (QM/QM′) method. Despite the similar potential energy surface (PES) profiles observed in their isolated forms, distinct differences emerged in the cluster model simulations of α-SA4B and β-SA4C. This observation suggests that the molecular environment influenced the torsional energy landscape associated with their chromic properties.

**Method:**

Each crystal was represented by a cluster model consisting of a central molecule and 14 peripheral molecules arranged based on crystallographic symmetry. The central molecule was treated as the high-level QM layer, while the surrounding molecules were kept fixed as the low-level QM′ layer. Following iterative optimization of the cluster model, the PES was calculated to track the *cis-trans* isomerization process of the central molecule. The iterative optimization was conducted with ONIOM (B3LYP/6-311G**: HF-D3BJ/6-31G*), and the PES was calculated with the same level. For each cluster model, the excited state was calculated with the TD-DFT method. All the quantum chemical calculations were performed using Gaussian 16 software.

**Graphical abstract:**

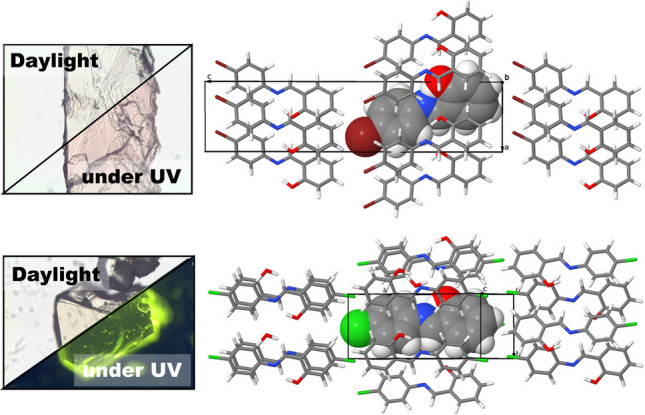

**Supplementary Information:**

The online version contains supplementary material available at 10.1007/s00894-025-06579-2.

## Introduction

**Fig. 1:**
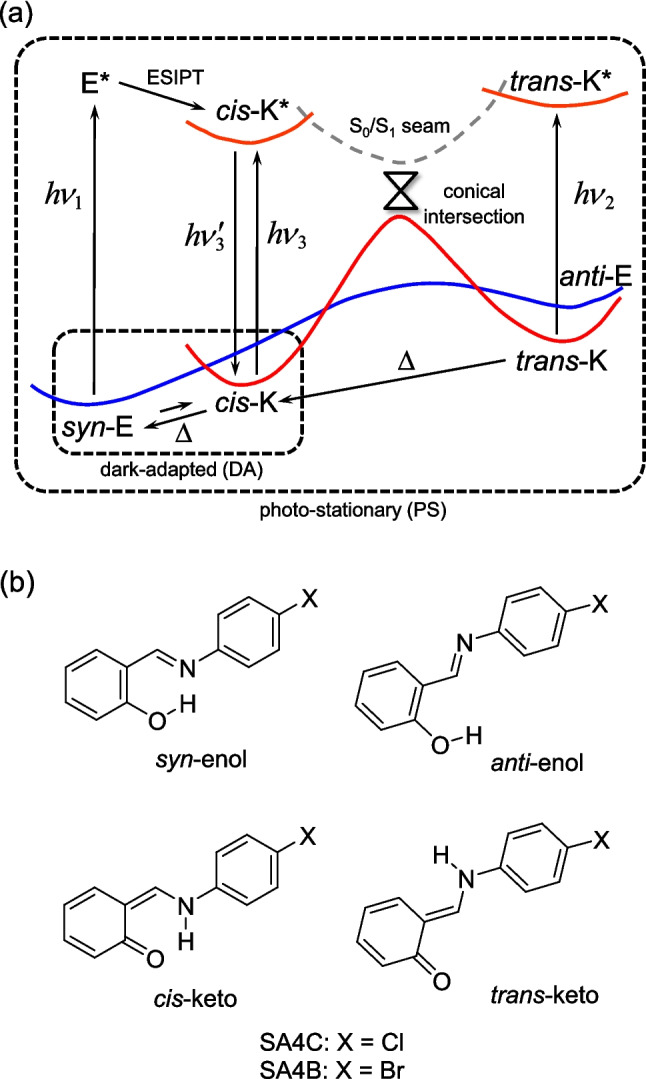
**a** Schematic representation of the widely accepted thermal and photochemical processes of SAs, where E and K denote the enol and keto forms, respectively. **b** Possible rotamers and tautomers of SA4C and SA4B

The chromic phenomenon is a stimulus-induced reversible color change that has garnered attention due to its potential applications in sensors, transducers, and signal attenuators [1]. Among various chromic materials, molecular crystals of salicylideneanilines (SAs) have been extensively studied for their photochromism and thermochromism [2]. Thermochromism arises from a thermal equilibrium bias between the enol and *cis*-keto tautomers at ambient temperature, while photochromism involves photoexcitation leading to excited-state proton transfer (ESIPT) and isomerization to the *trans*-keto tautomer (Fig. 1). Since the seminal work of Cohen and Schmidt [3], it has been observed that photochromic SA crystals consist of loosely packed nonplanar molecules, contrasting with thermochromic crystals that feature planar molecules closely packed in planes [4]. While this empirical rule applies to many SA crystals, exceptions are increasingly found in systems created through co-crystallization and salification [5–7]. The relationship between crystalline/molecular structures and thermochromism/photochromism remains unclear. With the discovery of more exceptions challenging traditional empirical rules, quantum chemical methods are becoming essential for elucidating fundamental principles.

In a recent study [8], we presented the unique chromic properties of two congeneric salicylideneaniline (SA) derivatives, *N*-salicylidene-4-bromoaniline (SA4B) and *N*-salicylidene-4-chloroaniline (SA4C). Previous reports indicated that despite their similar electronic structures, these molecules crystallize differently, resulting in distinct chromic behavior. In particular, SA4B in the α-form demonstrated simultaneous photochromism and thermochromism, while SA4C in the β-form exhibited only thermochromism. We prepared a series of solid solutions of these two compounds and demonstrated that, with certain compositions, SA4C molecules were integrated into the α-form, contributing to photochromic activity. This observation exemplifies that chromic properties are primarily influenced by molecular packing within the crystal rather than by the intrinsic nature of the molecule itself.

To delve deeper into the chronic properties, we sought to replicate the molecular environments present in the crystals of SA4B and SA4C through model chemistry. Our approach involved utilizing the Own N-layered Integrated molecular Orbital and Molecular mechanics (ONIOM) method [9, 10], a hierarchical hybrid QM/QM′ technique that combines various quantum chemistry methods for specific segments of a system. Widely utilized in exploring biomolecular systems, transition metal complexes, and catalysts, this method provides dependable geometric and energetic outcomes at a reasonable computational cost [9, 11–13]. The investigation of the ESIPT mechanism in SA and its derivatives has been a focal point in recent years [14–22]. However, the application of this approach in molecular crystal studies remains limited. To address computation efficiency, many studies have instead employed molecular mechanics/embedded mechanism techniques to model the crystal environment (surrounding molecules) [23–26], while other studies have utilized periodic density functional theory (DFT) methodology to produce the crystalline symmetry [11, 27, 28]. While generally suitable, prior studies encountered challenges in reproducing molecular geometric changes. A significant obstacle arises from the need to construct appropriate geometric inputs due to the occasional unavailability of precise structural data. These data play a critical role in energetically analyzing various processes such as prototropic tautomerization and *cis*-*trans* isomerization. Certain advanced programs can optimize the geometry of crystal structures within periodic boundary conditions, such as the VASP software package for predicting the mechanical properties of crystalline materials [29–32]. However, they do not address local molecular events that disrupt this periodicity.

To achieve a reasonable resolution, we adopted an iterative method to optimize a molecular cluster consisting of the central unit and its surroundings. A similar methodology was reported by Koibuchi et al. [33, 34]. This method provides a satisfactory approximation of the molecular environment within a specific crystal structure, effectively balancing precision and computational cost. Following the validation of the model’s reliability against experimental data, it will facilitate the computation of potential energy surface (PES) curves associated with the *cis*-*trans* isomerization of SA molecules in their crystalline forms. This enhances our comprehension of the correlation between crystal structure and chromic properties. In this investigation, we developed cluster models for SA4B and SA4C crystals, aiming to elucidate the structure–function relationship inherent in these crystals.

## Computational details

### Optimization of clusters

The initial structures of the α-SA4B and β-SA4C crystals were modeled based on single-crystal X-ray data from the CCDC (refcode 2193163 and 231864, respectively). While the α-form of the SA4C crystal is known, its kinetic instability inhibits the elucidation of the crystal structure. Hence, we approximated its cell constant using the crystal structures of a series of SA4[C_*x*_B_*y*_] solid solutions (vide infra). To maintain the original conformation in the crystal, we selected 15 molecules to form a cluster model comprising one central molecule and 14 peripheral molecules to fully cover the solvent-accessible surface of the central molecule. The solvent-accessible surface was defined as a sphere with a radius of 1.2 Å that touches the van der Waals volume of a molecule and was visualized using Jmol software [35]. This cluster, denoted as 0@0, signifies that the central molecule, after zero optimization cycles, is surrounded by molecules with the same geometry. The cluster structure was refined using the Gaussian16 software package [36]. The density functional and the basis sets employed were designated in each of the following procedures. We considered the empirical function of dispersion force by including Grimme’s D3BJ correction [37].

1. Iteration: The ONIOM (B3LYP/6-311G**: HF-D3BJ/6-31G*) method was specified with the “Opt” keyword to optimize the central molecule while keeping all surroundings frozen. Once the optimization converged, the geometry of the central molecule was copied and located at the position of the peripheral 14 molecules, so that the local crystalline symmetry is maintained within a fixed cell. This version of the cluster is denoted as 1@1. After optimizing 1@1, the cluster was renamed 2@1. The peripheral molecules were then substituted with the central molecule, resulting in the cluster being renamed as 2@2. This cycle was repeated until the total energy (extrapolated energy in the ONIOM scheme) of the *n*@(*n*−1) cluster was within 2 kJ mol^−1^ compared to the previous cycle ((*n*−1)@(*n*−2)). Additionally, we monitored the root mean square value of the atomic displacement (heavy atoms only) in the central molecule, as indicated by the initial CIF coordinates (0@0). The “Nosymm” keyword was utilized to maintain the coordinates relative to the unit cell for the entire cluster throughout each iteration.
2. Energy refinement: Once the reliable cluster model was obtained, the central molecule in the *n*@(*n*−1) cluster was calculated by full DFT calculations under B3LYP-D3BJ/6-311G** and ωB97XD conditions. The geometry was also used as input for normal-mode vibrational analysis for infrared (IR) spectral simulation at the ONIOM (B3LYP/6-311G**: HF-D3BJ/6-31G*) level.
3. Rotation tracking: The central molecule was adjusted to monitor the pedal motion relative to the Ar(salicyl)–C=N–Ar(aniline) moiety by altering specific geometric parameters (the structure was relaxed through preoptimization, if required). The molecule was inserted at the center of the *n*@(*n*−1) cluster and optimized under ONIOM (B3LYP/6-311G**: HF-D3BJ/6-31G*) conditions, maintaining φ_1_ (the dihedral angle of C(-OH)–C(ipso)–C = N) at a fixed value from − 180 to + 180° in 15° increments using the “ModRedundant” keyword. Similar tracking calculations were conducted for the keto tautomers by adjusting the proton position.
4. Excited state calculations: Using the above-mentioned geometry data as an input, the excited state of the central molecule was calculated with the time-dependent density functional theory (TD-DFT) calculation combined with the ONIOM method under “ONIOM (B3LYP/6-311G** TD: HF-D3BJ/6-31G*)” conditions. The S_1_ excited-state geometry was optimized with the constraint of the torsion angle, and the PES curves to track rotations were drawn similar to step (3).

### Modeling of putative crystal

**Fig. 2:**
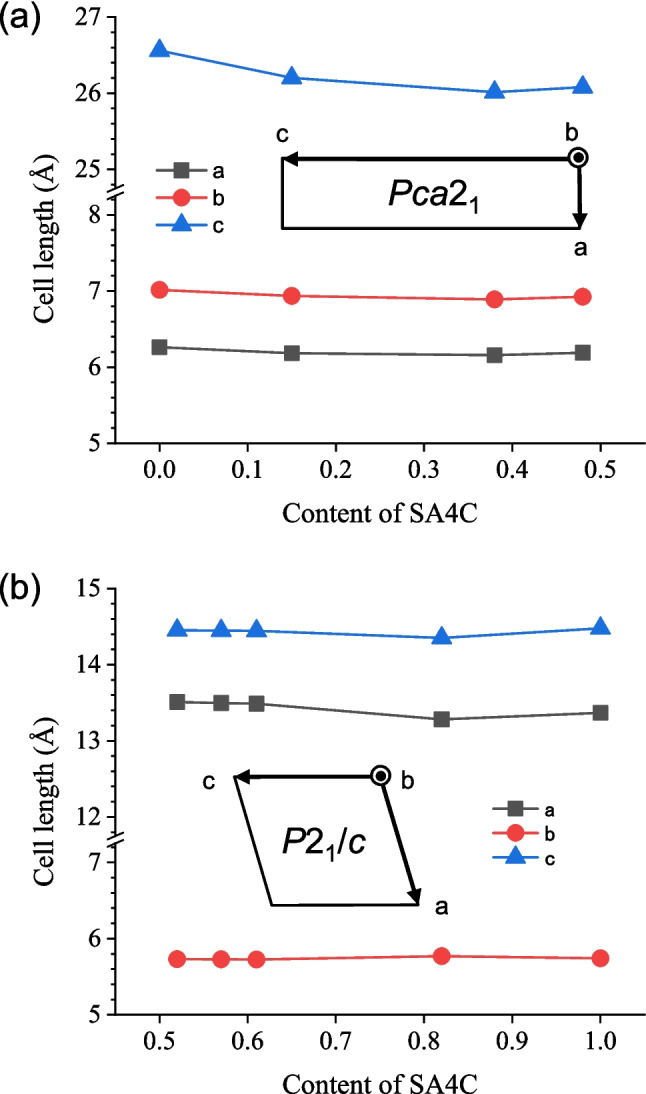
Cell constants for SA4[C_*x*_B_*y*_] solid solutions as a function of *x*, representing the content of SA4C. When 0 < *x* < 0.5, the crystal structure resembles α-SA4B (orthorhombic), while for 0.5 < *x* < 1.0, it resembles β-SA4C (monoclinic)

Recently, we demonstrated that an unstable polymorph of SA4C, exhibiting photochromism, is anticipated to possess a crystal structure similar to that of the α-SA4B crystal [8]. Subsequently, we refer to this kinetic phase as α-SA4C. We endeavored to develop a cluster model for SA4C using the same strategy as described above. Despite the unavailability of a single-crystal X-ray structure for the pure α-SA4C phase owing to its unstable nature, the crystal structures of various SA4[C_*x*_B_*y*_] solid solutions (SA4C: SA4B = *x*: *y*) have been elucidated. The crystal constants obtained are depicted in Fig. 2. Considering the gradual decrease in the *c*-axis length with increasing *x* (0 < *x* < 0.5) for α-type crystals (Fig. 2a) and the relatively stable cell constants for β-type crystals with *x* values ranging from 0.5 to 1.0 (Fig. 2b), selecting SA4[C_0.5_B_0.5_] as a potential cluster model for α-SA4C seemed reasonable. The chosen lattice parameters were *a* = 6.190 Å, *b* = 6.925 Å, and *c* = 26.08 Å, slightly smaller along the *c-*axis compared to those of α-SA4B (*a* = 6.1808 Å, *b* = 6.9398 Å, *c* = 26.2336 Å). The 0.15 Å difference in the *c*-axis aligns with the 0.17 Å variance in a typical bond length between C–Cl (1.77 Å) and C–Br (1.94 Å). Furthermore, the constructed α-SA4C cluster model was also optimized as described above and used as an initial structure for rotation tracking and excited-state calculations.

## Results

### Cluster modeling

To create cluster models of the SA4C and SA4B crystals, we utilized X-ray crystallographic data archived at the CCDC under refcodes 231864 (SA4C) and 2193163 (SA4B), the latter of which was recently reported by our group. While SA4C and SA4B are known to exhibit dimorphism, the available crystal structures are limited to their thermodynamically stable forms, specifically, the monoclinic *P*2_1_/*c* form (suffix β- in this study) for SA4C and the orthorhombic *Pca*2_1_ form (suffix α-) for SA4B. Rapid cooling of the β-SA4C melt affords α-SA4C, which shows photochromism but spontaneously turns to the β-phase at ambient temperatures. Therefore, in this work, we studied a putative cluster model for α-SA4C crystals as well as the known β-SA4C and α-SA4B crystals. Because the atomic positions in the XRD data have intrinsic measurement errors owing to statistical uncertainty, the cluster requires refinement before energetic evaluation.

**Fig. 3:**
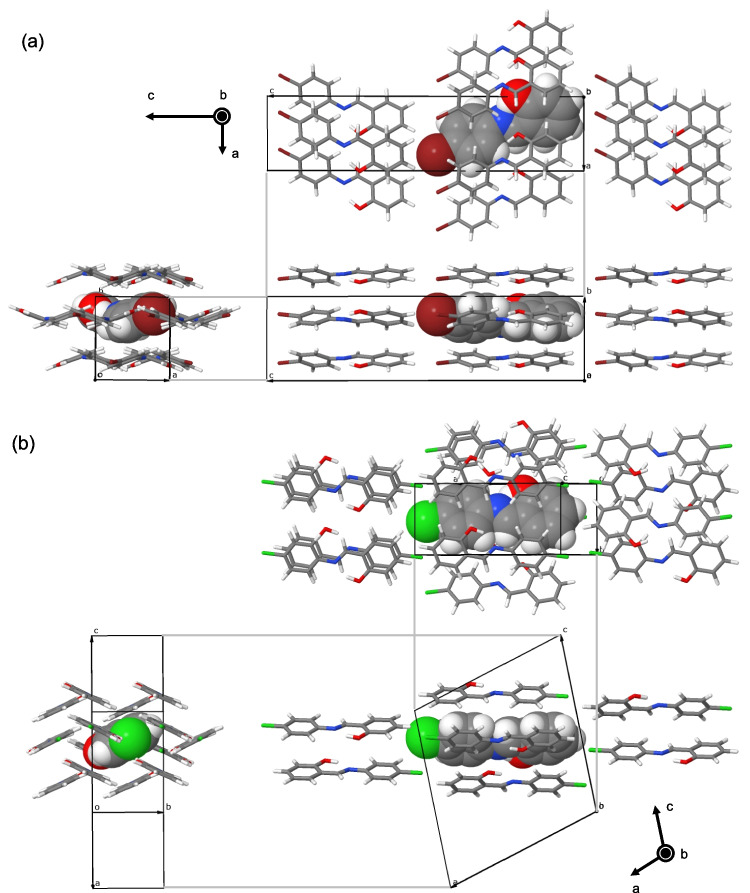
Molecular cluster models for **a** α-SA4B and **b** β-SA4C following an iterative geometric optimization. Views along three orthogonal directions are provided

Using the ONIOM method, we conducted iterative optimization of a cluster model consisting of 15 molecules to replicate the molecular structure and environment of the central molecule as observed in the crystal. The optimized clusters are shown in Fig. 3. The number (14) of surrounding molecules was chosen to adequately cover the solvent-accessible surface of the central molecule. While it was feasible to increase this number to include the second-nearest and third-nearest layers, in the initial phase of this study, we opted for the minimal model to capture the most significant influence of the molecular environment on the electronic state of the molecule under investigation. We believe that the calculation conditions used in this study are as stringent as those typically utilized in traditional quantum chemical studies. Nevertheless, the objective of this study is not to precisely replicate experimental measurements but rather to demonstrate the effective application of the ONIOM method in elucidating differences in molecular environments within crystals.

**Fig. 4:**
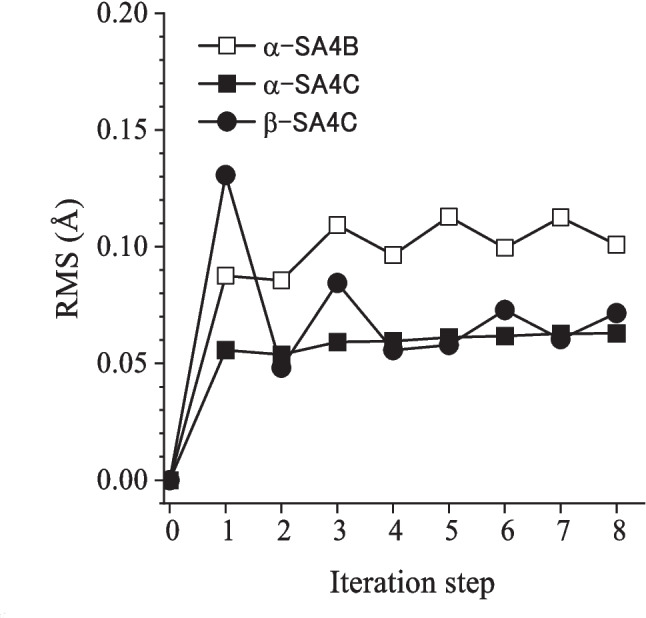
RMS values of atomic displacement from the initial crystal structure data plotted against the iteration step of the optimization process

The ONIOM energy (energy of the real system extrapolated to the high-level method) for each iteration step is summarized in Table 1 (see Table S1−S3 for full data). We compared the energies of subsequent cluster versions relative to that of 2@1. For α-SA4C, the energies of the 8@7 and 7@6 clusters were within 2 kJ/mol (0.13 kJ/mol per constituent molecule), a criterion established in previous work. For β-SA4C, the 7@6 clusters achieved this criterion. In the case of α-SA4B, although the relative energy exhibited oscillating behavior, which we could not deal with in the present framework, we truncated the iteration at the eighth step. The difference between 8@7 and 6@5 was only 0.4 kJ/mol; hence, we regarded the 8@7 cluster with sufficient convergence of the geometry. Throughout the iteration process, the overall geometry change was monitored by plotting the root mean square (RMS) values of the heavy atom displacements relative to the initial crystal data (Fig. 4). The RMS values did not exceed 0.1 Å (0.01 Å as standard error) after eight cycles, suggesting that the cluster maintained the original packing structure in the crystal. Consequently, the 8@7 cluster data were used for surrounding molecules in subsequent calculations.

**Table 1:** Comparison of energies (in kJ/mol, relative to 2@1 cluster) obtained through iterative ONIOM calculations (B3LYP/6-311G**: HF-D3BJ/6-31G*)

	4@3	5@4	6@5	7@6	8@7
α-SA4B	2.63	− 5.54	1.47	− 3.49	1.89
α-SA4C	0.11	3.23	1.89	2.18	2.42
β-SA4C	− 20.72	− 17.12	− 20.64	− 19.09	− 25.02

The central molecule was transformed into the keto form and optimized while surrounded by enol-form molecules, whose geometry remained fixed. This approach yields the energy values of the keto form compared to the enol form within the identical molecular environment in a specific crystal. This setup replicates a scenario where the likelihood of tautomerization is minimal, preventing the simultaneous transformation of the neighboring molecules.

After geometry optimization, the total energy of the cluster was refined using higher-level computational conditions: full DFT calculations under the B3LYP-D3BJ/6-311G** and ωB97XD/6-311G** conditions. Throughout these analyses, only the central molecule was optimized, while the surrounding molecules remained fixed within the cluster models.

Table 2 compares the energies of the clusters in the enol- and *cis*-keto forms; the proton transfer energy (Δ_PT_*E*) is the difference between the two. To elucidate the impact of the molecular environment on the chromic properties, comparing the Δ_PT_*E* is more meaningful than discussing individual tautomer energies. Because the ONIOM scheme did not allow the simultaneous use of the empirical dispersion correction over the multi-layers, we chose to apply the correction to the low-layer HF method. Indeed, the DFT-D method applied to the central molecule had minimal influence on the energy difference within the ONIOM scheme. This difference in impact is attributed to boundary issues inherent in ONIOM calculations, i.e., the interaction energy within the whole system is calculated with the low-layer method. Additionally, using the full DFT method, which offers theoretically more precise results albeit requiring considerably longer computation times, yielded similar energy differences between the enol- and *cis*-keto tautomers for the same crystal form (α or β). This consistency was also observed using the ONIOM method, with both methods showing an α- and β-form energy difference of 3–4 kJ/mol.

**Table 2:** Energies of the clusters in OH- and *cis*-NH-forms, along with the proton transfer energy (Δ_PT_*E* in kJ mol^−1^). Comparison among ONIOM and full DFT calculations

	ONIOM (DFT: HF-D)	Full DFT-D	
	B3LYP/6-311G**: HF-D3BJ/6-31G*	B3LYP-D3BJ/6-311G**	B97XD/6-311G**
α-SA4B	13.35	14.68	18.41
α-SA4C	13.17	14.37	18.09
β-SA4C	9.89	11.14	14.79

### Reliability test

The acquired cluster model facilitates the normal-mode vibration analysis of the central molecule, even if it is not the energy-minimum structure in the isolated state. Comparing the IR spectra between the observed one and that expected from the normal-mode analysis serves as a reliable measure for evaluating the quality of the computational models. Various factors influence the IR spectra in the crystalline state compared to the isolated state, such as (1) specific intermolecular interactions like hydrogen bonding that can significantly modify the nature of chemical bonds, (2) packing effects that force molecules to assume a conformation different from their isolated state, and (3) coupling of the vibrational modes among neighboring molecules. In this instance, we can largely disregard factor (1) since no such interactions were observed in the crystal structures of SA4C and SA4B. Factor (2) is the most probable cause, as the SA4C molecules in the crystal exhibit a planar structure, while SA molecules typically favor a twisted conformation. For the isolated SA4C molecule, the C=N–C=C dihedral angle was optimized at 37.1°, whereas in the β-SA4C cluster, the angle was 14.3°, closely resembling the 5.8° angle found in the corresponding crystal structure. Factor (3), which results in peak broadening due to phonon dispersion, is anticipated to be minimal in the mid-IR region or higher. The correlation of the mass-weighted atomic displacement vectors between the molecules in the cluster and in the vacuum forms an almost linear relationship (Fig. S1 and S2), indicating limited coupling between the vibration modes mediated by the molecular environment in the crystal [38].

**Fig. 5:**
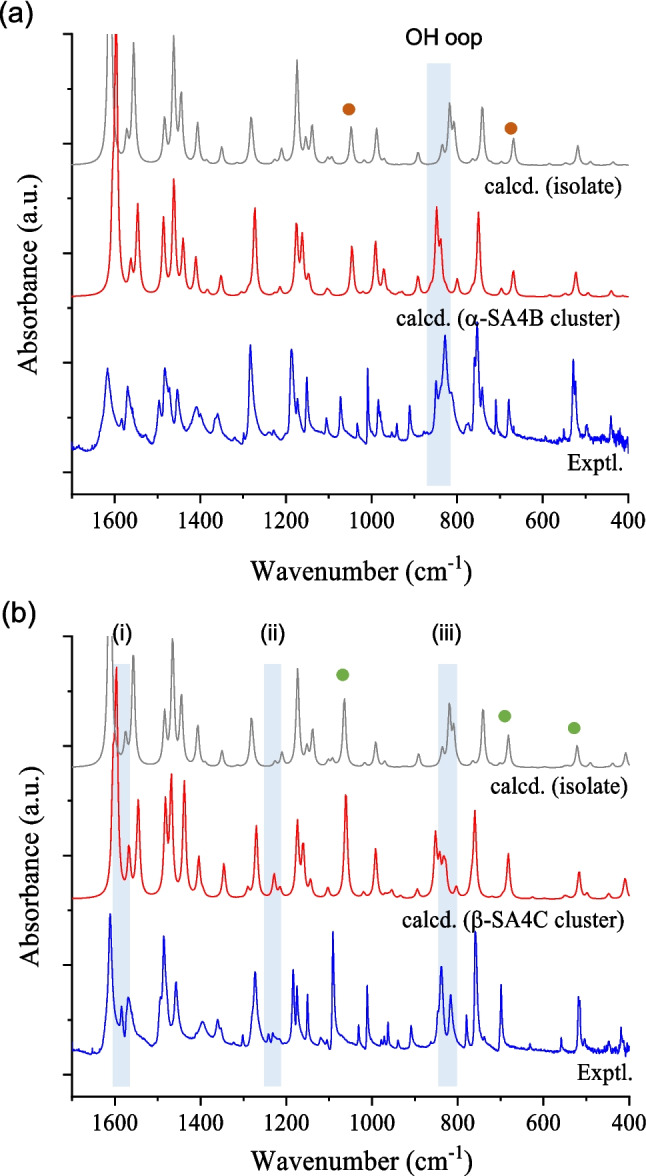
Comparison of IR spectra calculated and observed for **a** α-SA4B and **b** β-SA4C: calculated spectra of the isolated molecule (gray lines), the central molecule in the cluster (red lines), and experimental spectra (blue lines). Vibration peaks associated with C–Br and C–Cl bonds are indicated by brown and green dots, respectively. Refer to the text for the bands identified as (i)–(iii)

Our recent effort to calculate the IR spectra for molecular clusters showed moderate agreement with the observed spectra, likely due to considering conformation effects. Figure 5 compares the theoretical simulations for the isolated molecule, the molecule in the cluster (8@7), and the observed spectra for both β-SA4C and α-SA4B. All calculated wavenumbers are listed in Table S4, along with the assignments of observed peaks. As anticipated, the calculated IR spectra of isolated SA4B and SA4C molecules were similar to each other. The main difference was a systematic shift resulting from the difference between C–Br and C–Cl bonds: the modes involving C–Br stretching were 1047.3, 668.4, 346.4 cm^−1^, corresponding to the C–Cl counterparts at 1064.7, 681.9, 408.3 cm^−1^. For the α-SA4B cluster, the calculated peaks were relatively consistent between the cluster and isolated states, reflecting the similarity in their conformations. These calculated peaks also align well with the observed peaks. One noticeable discrepancy lies in the pair of 807.0 cm^−1^ for the isolated state and 847.9 cm^−1^ for the cluster, which was identified as 849 cm^−1^ in the observed spectrum. This discrepancy is attributed to the out-of-plane bending of the Ar–O–H moiety, influenced by the neighboring molecules in the cluster.

The spectra calculated for the β-SA4C cluster model moderately reproduced the observed peaks but exhibited a slight deviation from the isolated molecule. Specifically, weak absorption peaks at 1567.2, 1228.4, and 803.4 cm^−1^ were prominent only in the β-SA4C cluster calculation data, potentially corresponding to peaks at 1585, 1241, and 816 cm^−1^ in the observed spectrum. These peaks were identified as (i) coupled in-plane distortion of the salicylidene and aniline rings, (ii) coupled C–H bending of the aromatic rings and azomethine group, and (iii) out-of-plane bending localized at the aniline ring. Notably, the 803.4 cm^−1^ peak calculated for the cluster was significantly absent in the isolated molecule, likely due to conformational changes influenced by different molecular environments. Despite some assignment uncertainty, the overall profiles showed good agreement between the calculated and observed spectra. The results of the reliability test indicate that the cluster model obtained can facilitate comparisons of the molecular environments of the SA4C and SA4B crystals.

### PES study for pedal motion

Conventional physical chemistry studies of salicylideneanilines [39–41] offer insights into the transformation of the SA molecule between *cis*- and *trans*-forms concerning the iminomethyl-2-phenol moiety (C(–OH)–C(ipso)–C=N) during photocoloration and bleaching processes. While the torsion angle of the aniline moiety has no constraint in the isolated state, it must change in concert with the *cis*-*trans* isomerization in the restricted space of the crystalline environment. Consequently, the Ar–C=N–Ar moiety exhibits a bicycle-pedal-like motion, known as pedal motion, indicating the necessity to monitor not only the torsion angle but also the overall conformational changes of the central molecule. Owing to this complexity, a standard PES scan protocol was unsuitable, necessitating the preparation of initial structures at various isomerization stages, each requiring full optimization to align with the crystal field. Analyzing the PES concerning the pedal motion relevant to *cis-trans* isomerization would reveal variations in the molecular environment, offering insights to elucidate the differing chromic functionalities among the allomorphic crystals.

A set of calculations was applied to the keto and enol forms in the crystal environment (cluster models of α-SA4B, α-SA4C, and β-SA4C) as well as in the isolated state. The methodology for determining the PES for the pedal motion is outlined in the calculation section. A 360° PES scan was performed under ONIOM (B3LYP/6-311G**: HF-D3BJ/6-31G*) conditions for the structure of the central molecule, with a fixed C(–OH)–C(ipso)–C=N angle and the optimized surroundings as mentioned above. This setup assumes that the enol-keto tautomerization and pedal motion have a sufficiently low probability, preventing neighboring molecules from undergoing simultaneous transformations in the crystal lattice. The calculated “high model” energy is for the central molecule suffering conformational constraints in the crystals. Comparing the torsion angle dependence of the high model layer with that of the isolated molecule (Fig. S4), there was a small but non-negligible discrepancy attributable to the conformation of the aniline moiety, which may also alter the intermolecular interactions that affect the overall system energy.

**Fig. 6:**
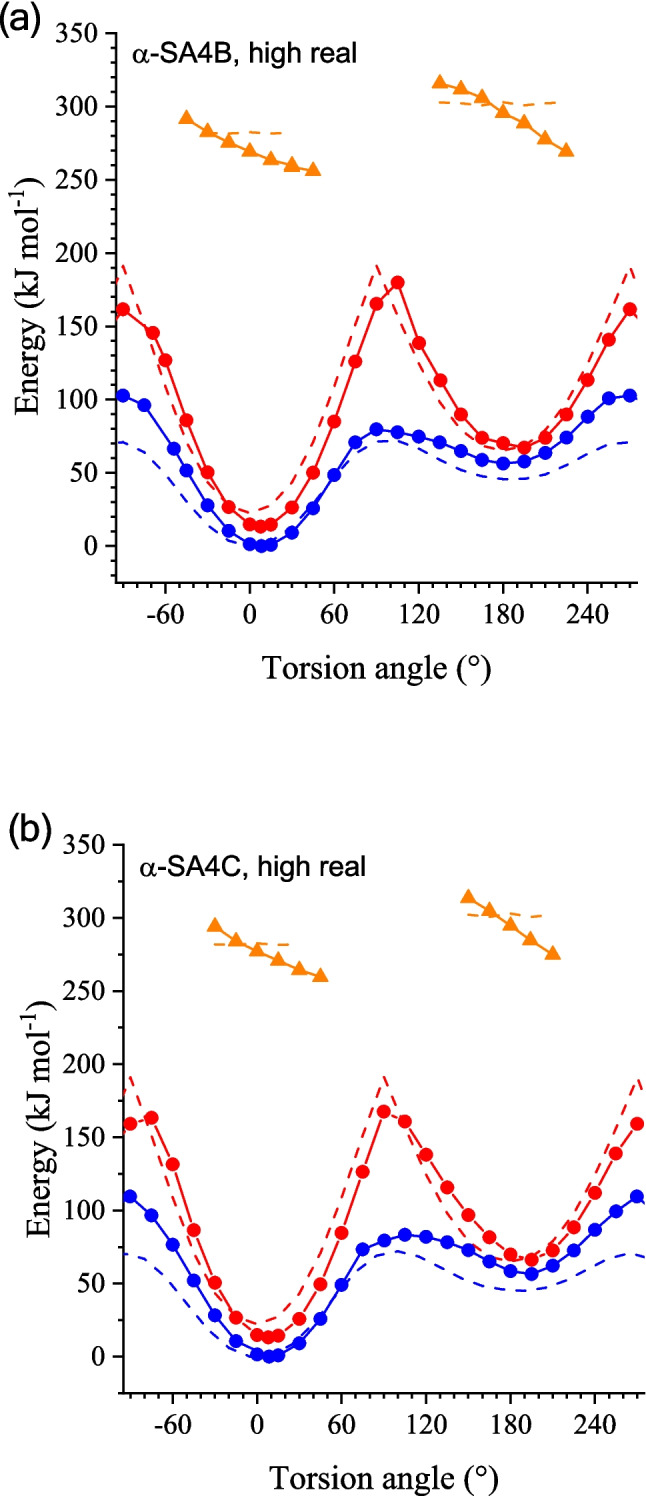

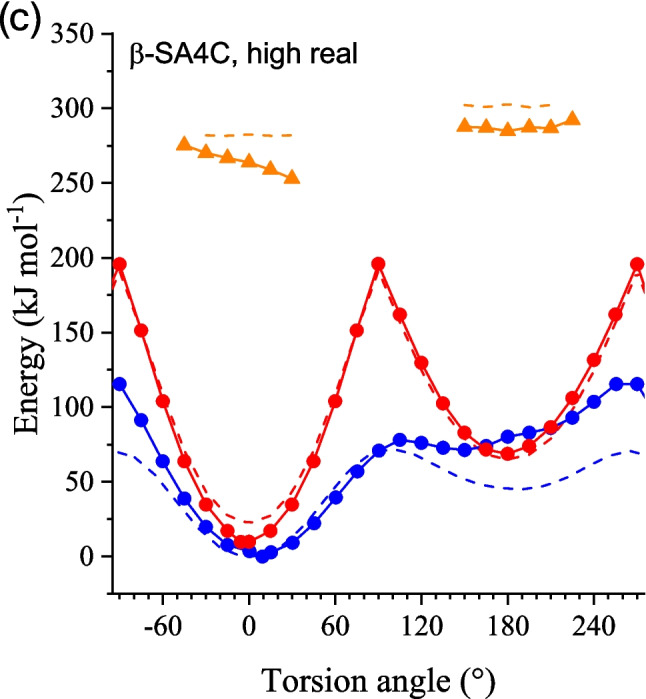
Ground-state PES curves of the enol- (blue lines) and keto-forms (red lines) relevant to the pedal motion involved in the *cis*-*trans* isomerization. Solid lines are those calculated for cluster models of **a** α-SA4B, **b** α-SA4C, and **c** β-SA4C, and dashed lines are those calculated for the isolated state. Excited (S_1_)-state PES curves of the keto-forms (orange solid/dashed lines) are also shown

Figure 6 displays the PES curves of the three clusters in the ground state and the S_1_ excited state within the range from − 90 to 270° of the torsion angles. As is seen in Fig. 6a and b, the overall profiles of both the enol (blue solid lines) and keto forms (red solid lines) are similar between α-SA4B and α-SA4C. In the enol form, two energy minima were observed around 0° (*syn*-enol) and 180° (*anti*-enol). Comparison with the corresponding isolated molecules (dashed lines) indicated that the crystal environment destabilizes the *anti*-form by about 10 kJ mol^−1^. The PES curves for the keto form also displayed two energy minima around 0° (*cis*-keto) and 180° (*trans*-keto), between which there is an energy barrier for transition state around 90°. The energies of *cis*-keto (*E*_*cis*K_), *trans*-keto (*E*_*trans*K_), and the transition state (*E*_TS_) were 13.4, 67.2, and 179.9 kJ mol^−1^ (referenced by *syn*-enol), respectively, for α-SA4B, while they were 13.2, 66.3, and 163.4 kJ mol^−1^ for α-SA4C.

In contrast to the similarity between the α-form crystals, the β-SA4C crystal cluster exhibited distinct PES curves for both the enol and keto forms (Fig. 6c). The enforced planar configuration of the SA4C molecules in the β-form crystals primarily contributed to the higher energy of the high model part compared to the isolated molecule. For the enol form, the energy minimum around 180° was relatively shallow and more destabilized (80 kJ mol^−1^ vs. *syn*-enol) compared to α-SA4C. In contrast, the PES curve for the keto form resembled that of the α-form crystals but featured deeper potential wells: the energies of *cis-*keto (*E*_*cis*K_), *trans*-keto (*E*_*trans*K_), and the barrier top (*E*_TS_) were 9.8, 68.8, and 195.9 kJ mol^−1^, respectively. The modest energy difference (9.8 kJ mol^−1^) of *E*_*cis*K_ aligned with the thermochromic behavior of β-SA4C, while the values (13.4 and 13.2 kJ mol^−1^) for α-SA4B and α-SA4C crystals were also consistent with their thermochromism. These differences were notably smaller than those (22.7 kJ mol^−1^ for SA4B and 22.8 kJ mol^−1^ for SA4C) of the isolated molecule, indicating the effective induction of thermochromism by the crystalline environment.

Interestingly, the ground-state PES profiles of the α-SA4B and α-SA4C crystals resembled those of the photochromic α_1_-form of the SA crystal, while the β-SA4C crystal’s profile was similar to that of the thermochromic β-SA crystal [33]. Ground-state PES is valuable for understanding back-proton transfer during bleaching after photoisomerization. The bleaching of the photocolored species (*trans*-keto form) can proceed through thermal relaxation (T-type) or photochemical relaxation (P-type). We recently devised an experimental procedure to determine the kinetic parameters for these processes separately. Depending on molecular and crystal structures, these parameters influence bleaching differently. Our observations indicate that the α_1_-SA and α_2_-SA crystals primarily bleached via a photochemical pathway, suggesting that the activation barrier (~ 100 kJ mol^−1^) for back-isomerization from *trans*-keto to *cis*-keto forms was sufficiently high to inhibit thermal bleaching. Our calculations demonstrated that the activation barrier exceeded 95 kJ mol^−1^, suggesting that the α-form SA4B/SA4C crystals would exhibit P-type photochromism.

The ground-state PES mentioned above does not directly provide insights into the photochemical process involved in the photochromism of SAs. These processes involve ESIPT and a pathway through the minimum energy conical intersection (MECI) around a 90° torsion angle, as reported by various researchers [17, 42–44]. TD-DFT is widely recognized as the most suitable method for calculating excited-state energies with moderate accuracy when studying ESIPT-related organic species [45–48]. The excited-state calculation of large systems like proteins and molecular clusters presents an intrinsic difficulty, while rigorous but high-cost computational methods are developed by some groups [49–52]. Meanwhile, the combination of ONIOM and TD-DFT is regarded as a practical and reasonable choice to do excited-state analysis for the high-layer molecule. As per our current purpose, we have chosen to employ this scheme as a potential method to evaluate the excited state of the central molecule within the molecular environment. As a result, the calculation led to a conformation different from that of the isolated molecule.

In this study, we confined the application of this method to relaxed S_1_ states of the keto form to draw excited-state PES and gain insight into the excited-state dynamics relevant to photochromic behavior. Figure 6 shows the PES curves (orange solid lines) of the S_1_ excited state for the three-cluster models. The structures of the self-consistent cluster model (8@7) of α-SA4B, α-SA4C, and β-SA4C served as the initial geometry data for the pedal motion study. The dihedral angle of Ar–C=N–Ar was fixed using the opt=modredundant command for the relaxed PES scan. Similar to prior studies employing quantum chemistry methods for S_1_ structure calculations, TD-DFT calculations under Gaussian conditions did not converge near the MECI. Consequently, data on the relaxed S_1_ state lacks information within approximately ± 15° around the perpendicularly twisted conformation.

Despite some limitations, these PES curves exhibit noticeable distinctions between α- and β-form crystals: the α-SA4B and α-SA4C results display a significant decrease from − 45 to + 45° for the *cis*-keto form and from + 135 to + 225° for the *trans*-keto form, while the β-SA4C result exhibits less steep curves. For instance, the energy decreases from − 30 to 30° were 30 and 17 kJ/mol for α-SA4C and β-SA4C, respectively. These tendencies are also reminiscent of our previous report on non-substituted SA crystals [33], from which we concluded that for photochromic α_1_-SA and α_2_-SA polymorphs, the S_1_-state PES curves were inclined so that the molecule could be prone to twist toward the MECI, whereas, for non-photochromic β-SA polymorph, the curve is rather flat so that the molecule can stay planar, resulting in ESIPT emission. These similarities in the curve appearance are in line with the observation that α-SA4B and α-SA4C are photochromic and β-SA4C is non-photochromic.

## Discussion

While the quantum chemistry approach to the structure–function relationship of molecular crystals is promising, it can be costly. In this study, we utilized molecular cluster models to mimic the molecular environment within a specific crystal. Recently, we applied the same strategy to explain the differences in chromic behavior among three polymorphs of salicylideneaniline. We obtained meaningful insights into the relationship between PES and chromic properties, although the results need to be extensively verified for a wide variety of crystals of similar compounds.

SA crystals exhibit various polymorphs, each with distinct properties such as photochromism, thermochromism, fluorescence, and phosphorescence. Although rigorous quantum chemical studies of single molecules have been conducted to clarify the mechanisms behind these features, single-molecule or solution principles are often not applicable to solid-state situations. Therefore, developing a model to simulate the crystal environment is crucial. This field has been well studied, with MD and QM/MM using periodic DFT calculations [53, 54] becoming popular for solid-state simulations. However, because the mechanism of certain functionalities often involves molecular structural transformations or even photochemical processes, any large-scale targeting approach is simply not capable of accurately describing these changes. According to previous single-molecule studies, the photochromism of SA comprises a series of local molecular events such as photoexcitation, ESIPT, and an imine bond rotation of approximately 90° to reach the MECI.

Combining all the issues mentioned above, the cluster model study of SAs is a significant trial in the structure–function study of molecular crystals. Using the available CIF files, we performed a Hirshfeld surface analysis [55] on the three crystals using CrystalExplorer software [56] to investigate the influence of surface interactions on the formation of these different crystal types. The corresponding results are shown in Fig. S5. As expected, the crystal fingerprints of the α-form crystals were similar. In contrast, the fingerprint of β-SA4C appeared noticeably different, although the area ratios of the atom–atom contacts were not significantly different from those of α-SA4C. Careful comparison revealed that the molecules in the α-SA4C crystal have a greater surface area ratio of Cl–Cl contacts compared to those in the β-SA4C crystal. This suggests that halogen bonding may play a role in the differences in the crystal environment, thereby causing differences in the PES curves.

## Conclusion

In this study, we present a computational chemistry investigation using cluster models to elucidate the mechanism of three crystals: α-SA4B, α-SA4C, and β-SA4C, exhibiting distinct chromic behaviors. Through our in-house modeling software, we conducted iterative geometry optimization, establishing that the cluster model generated a central molecule with intermolecular interactions mimicking the crystalline environment effectively. Consequently, this cluster accurately replicated the properties of SA4C and SA4B molecular crystals via IR spectral analysis. Despite the minimal size of the cluster model utilized in this study, it effectively captured the features of constrained molecular motion during *cis*-*trans* isomerization within a specific crystal environment. The PES curves offer insights into the energy variations induced by the crystal environment on both the enol and keto forms in the S_1_ excited state and the ground state. Interestingly, the comprehensive PES scan profile of the α-SA4B and α-SA4C crystals was similar to that of the photochromic α_1_-SA, whereas that of β-SA4C was similar to that of the thermochromic β-SA, implying that the cluster model can explain the structure–function relationship among different crystal structures. The present results suggest the importance of considering the interplay between the molecular conformation and crystal environment, neither solely one of them nor a simple sum of the two factors. In addition, reliable computational models for the relaxed S_1_ state are required for future photochemical studies, especially those focusing on the structure around MECI.

## Supplementary Information

Below is the link to the electronic supplementary material.

ESM 1 (DOCX 1.29 MB)

## Data Availability

Supporting information available: ONIOM energy of the iterative optimization process for the three polymorphic clusters, comparison of the calculated and experimental IR vibrational modes, correlation maps for vibration modes of isolated and in-cluster molecules, and some other calculated results are provided.
