# Supplementary material for: Modeling molecular environment of allomorphic N-salicylidene-4-halo-aniline crystals through iterative QM/QM′ structural optimization
Source: J Mol Model. 2025 Nov 27;31(12):351. doi: 10.1007/s00894-025-06579-2 (PMC12660421; doi:10.1007/s00894-025-06579-2)
Supplement: Supplementary file 1 — (DOCX 1.29 MB) [file 894_2025_6579_MOESM1_ESM.docx]

**Supporting information**

**Contents**

**Table S1** ONIOM optimized energy of α-SA4B cluster for each iteration step.

**Table S2** ONIOM optimized energy of α-SA4C cluster for each iteration step.

**Table S3** ONIOM optimized energy of β-SA4C cluster for each iteration step.

**Table S4** Vibration analysis for SA4C and SA4B (scaling factor = 0.967)

**Figure S1** α-SA4B vibration-mode correlation between isolated/in cluster molecules

**Figure S2** β-SA4C vibration-mode correlation between isolated/in cluster molecules.

**Figure S3** The comparison of calculated IR result for α-SA4C, β-SA4C and α-SA4B based on ONIOM cluster model.

**Figure S4** PES scan result for the high-layer in the cluster models.

**Figure S5** Hirshfield analysis of SA4X series crystals.

**Table S1** ONIOM optimized energy of α-SA4B cluster for each iteration step.

| SA4B_alpha |  |  |  |  |  |  |
| --- | --- | --- | --- | --- | --- | --- |
|  |  | Low_model | High_model | Low_real | High_real | High_real relative to 2@1 |
| [iter1](mailto:0@0) | [0@0](mailto:0@0) | -3197.380492 | -3205.517198 | -47961.24684 | -47969.38355 |  |
|  | [1@0](mailto:1@0) | -3197.540301 | -3205.685309 | -47961.40623 | -47969.55124 | 5869.04 |
|  |  |  |  |  |  |  |
| [iter2](mailto:0@0) | [1@1](mailto:1@1) | -3197.540341 | -3205.685307 | -47963.64141 | -47971.78638 |  |
|  | [2@1](mailto:2@1) | -3197.540514 | -3205.685257 | -47963.6419 | -47971.78664 | (0.00) |
|  |  |  |  |  |  |  |
| iter3 | [2@2](mailto:2@2) | -3197.540512 | -3205.685248 | -47963.64522 | -47971.78995 |  |
|  | [3@2](mailto:3@2) | -3197.540128 | -3205.685096 | -47963.64519 | -47971.79016 | -9.24 |
|  |  |  |  |  |  |  |
| iter4 | [3@3](mailto:3@3) | -3197.540113 | -3205.685115 | -47963.64046 | -47971.78546 |  |
|  | [4@3](mailto:4@3) | -3197.540299 | -3205.685126 | -47963.64081 | -47971.78564 | 2.63 |
|  |  |  |  |  |  |  |
| iter5 | [4@4](mailto:4@4) | -3197.540329 | -3205.685118 | -47963.64382 | -47971.78861 |  |
|  | [5@4](mailto:5@4) | -3197.540092 | -3205.685064 | -47963.64378 | -47971.78875 | -5.54 |
|  |  |  |  |  |  |  |
| iter6 | [5@5](mailto:5@5) | -3197.540095 | -3205.685052 | -47963.641 | -47971.78596 |  |
|  | [6@5](mailto:6@5) | -3197.540248 | -3205.685103 | -47963.64123 | -47971.78608 | 1.47 |
|  |  |  |  |  |  |  |
| iter7 | [6@6](mailto:6@6) | -3197.540269 | -3205.68511 | -47963.64302 | -47971.78786 |  |
|  | [7@6](mailto:7@6) | -3197.540098 | -3205.685061 | -47963.643 | -47971.78797 | -3.49 |
|  |  |  |  |  |  |  |
| iter8 | [7@7](mailto:7@7) | -3197.540095 | -3205.685064 | -47963.64087 | -47971.78583 |  |
|  | [8@7](mailto:8@7) | -3197.540214 | -3205.685089 | -47963.64105 | -47971.78592 | 1.89 |
| iter N | [N@7](mailto:N@7) | -3197.504294 | -3205.656518 | -47963.61391 | -47971.76613 |  |
|  | [N+@7](mailto:N+@7) | -3197.525927 | -3205.675235 | -47963.63153 | -47971.78084 | 15.23 |

**Table S2** ONIOM optimized energy of α-SA4C cluster for each iteration step.

| SA4C_alpha |  |  |  |  |  |  |
| --- | --- | --- | --- | --- | --- | --- |
|  |  | Low_model  (hartree) | High_model  (hartree) | Low_real  (hartree) | High_real  (hartree) | High_real relative to 2@1 (kJ/mol) |
| [iter1](mailto:0@0) | [0@0](mailto:0@0) | -1087.00394 | -1091.624611 | -16305.52272 | -16310.14339 |  |
|  | [1@0](mailto:1@0) | -1087.132829 | -1091.765047 | -16305.65074 | -16310.28296 | 4718.71 |
|  |  |  |  |  |  |  |
| [iter2](mailto:0@0) | [1@1](mailto:1@1) | -1087.132811 | -1091.765045 | -16307.44786 | -16312.08009 |  |
|  | [2@1](mailto:2@1) | -1087.132671 | -1091.764907 | -16307.44798 | -16312.08022 | (0.00) |
|  |  |  |  |  |  |  |
| iter3 | [2@2](mailto:2@2) | -1087.132731 | -1091.76491 | -16307.44837 | -16312.08055 |  |
|  | [3@2](mailto:3@2) | -1087.132668 | -1091.764898 | -16307.44836 | -16312.08059 | -0.97 |
|  |  |  |  |  |  |  |
| iter4 | [3@3](mailto:3@3) | -1087.13268 | -1091.764903 | -16307.44793 | -16312.08016 |  |
|  | [4@3](mailto:4@3) | -1087.13264 | -1091.764889 | -16307.44793 | -16312.08018 | 0.11 |
|  |  |  |  |  |  |  |
| iter5 | [4@4](mailto:4@4) | -1087.132577 | -1091.764874 | -16307.44668 | -16312.07897 |  |
|  | [5@4](mailto:5@4) | -1087.132632 | -1091.764881 | -16307.44674 | -16312.07899 | 3.23 |
|  |  |  |  |  |  |  |
| iter6 | [5@5](mailto:5@5) | -1087.132622 | -1091.764886 | -16307.44723 | -16312.07949 |  |
|  | [6@5](mailto:6@5) | -1087.132627 | -1091.764884 | -16307.44724 | -16312.0795 | 1.89 |
|  |  |  |  |  |  |  |
| iter7 | [6@6](mailto:6@6) | -1087.132604 | -1091.764882 | -16307.44711 | -16312.07939 |  |
|  | [7@6](mailto:7@6) | -1087.132631 | -1091.764882 | -16307.44714 | -16312.07939 | 2.18 |
|  |  |  |  |  |  |  |
| iter8 | [7@7](mailto:7@7) | -1087.132624 | -1091.764874 | -16307.44704 | -16312.07929 |  |
|  | [8@7](mailto:8@7) | -1087.132614 | -1091.764878 | -16307.44703 | -16312.0793 | 2.42 |
| iter N | [N@7](mailto:N@7) | -1087.097554 | -1091.736723 | -16307.42088 | -16312.06005 |  |
|  | [N+@7](mailto:N+@7) | -1087.118079 | -1091.754893 | -16307.43747 | -16312.07428 | 15.60 |

**Table S3** ONIOM optimized energy of β-SA4C cluster for each iteration step.

| SA4C_beta |  |  |  |  |  |  |
| --- | --- | --- | --- | --- | --- | --- |
|  |  | Low_model  (hartree) | High_model  (hartree) | Low_real  (hartree) | High_real  (hartree) | High_real relative to 2@1 (kJ/mol) |
| [iter1](mailto:0@0) | [0@0](mailto:0@0) | -1086.934472 | -1091.542910 | -16304.46263 | -16309.07107 |  |
|  | [1@0](mailto:1@0) | -1087.132590 | -1091.764598 | -16304.66192 | -16309.29393 | 7260.19 |
|  |  |  |  |  |  |  |
| [iter2](mailto:0@0) | [1@1](mailto:1@1) | -1087.132535 | -1091.764581 | -16307.42614 | -16312.05819 |  |
|  | [2@1](mailto:2@1) | -1087.132716 | -1091.764328 | -16307.42758 | -16312.05919 | 0.00 |
|  |  |  |  |  |  |  |
| iter3 | [2@2](mailto:2@2) | -1087.132721 | -1091.764331 | -16307.43627 | -16312.06788 |  |
|  | [3@2](mailto:3@2) | -1087.132662 | -1091.764348 | -16307.43662 | -16312.06830 | -23.92 |
|  |  |  |  |  |  |  |
| iter4 | [3@3](mailto:3@3) | -1087.132644 | -1091.764352 | -16307.43514 | -16312.06685 |  |
|  | [4@3](mailto:4@3) | -1087.132538 | -1091.764284 | -16307.43533 | -16312.06708 | -20.72 |
|  |  |  |  |  |  |  |
| iter5 | [4@4](mailto:4@4) | -1087.132547 | -1091.764287 | -16307.43388 | -16312.06562 |  |
|  | [5@4](mailto:5@4) | -1087.132688 | -1091.764293 | -16307.43411 | -16312.06571 | -17.12 |
|  |  |  |  |  |  |  |
| iter6 | [5@5](mailto:5@5) | -1087.132547 | -1091.764289 | -16307.43520 | -16312.06694 |  |
|  | [6@5](mailto:6@5) | -1087.132594 | -1091.764286 | -16307.43536 | -16312.06705 | -20.64 |
|  |  |  |  |  |  |  |
| iter7 | [6@6](mailto:6@6) | -1087.132608 | -1091.764266 | -16307.43473 | -16312.06639 |  |
|  | [7@6](mailto:7@6) | -1087.132638 | -1091.764275 | -16307.43482 | -16312.06646 | -19.09 |
|  |  |  |  |  |  |  |
| iter8 | [7@7](mailto:7@7) | -1087.132607 | -1091.764269 | -16307.43701 | -16312.06868 |  |
|  | [8@7](mailto:8@7) | -1087.132578 | -1091.76426 | -16307.43704 | -16312.06872 | -25.02 |
| iter N | [N@7](mailto:N@7) | -1087.099179 | -1091.738076 | -16307.41112 | -16312.05001 |  |
|  | [N+@7](mailto:N+@7) | -1087.120734 | -1091.756729 | -16307.42896 | -16312.06496 | -15.15 |

**Table S4**. Vibration analysis for SA4C and SA4B (scaling factor = 0.967)

|  | SA4C | | | SA4B | | |
| --- | --- | --- | --- | --- | --- | --- |
|  | isolated | in cluster | obsd. | isolated | in cluster | obsd. |
| rd(sa)+ip(OH) | 1612.8 | 1604.1 | 1611 s | 1612.6 | 1604.6 | 1617 s |
| str(CN) | 1610 | 1596.4 |  | 1609.7 | 1596.5 |  |
| rd(sa+an) | 1575.1 | 1567.2 | 1585 m | 1571.7 | 1562.3 | 1584 w |
| rd(sa+an) | 1557 | 1550.4 | 1568 m | 1555.6 | 1546.2 | 1570 s |
| rd(sa+an)+ip(OH) | 1483.9 | 1481.9 | 1493 sh. | 1483.3 | 1485.9 | 1496 s |
| rd(an) | 1465.2 | 1468.4 | 1486 s | 1462 | 1461.5 | 1483 s |
| rd(sa) | 1444.8 | 1438 | 1457 m | 1444.7 | 1434 | 1453 s |
| rd(sa) | 1406.6 | 1404.2 | 1396 m | 1406.3 | 1410.3 | 1408 m |
| ip(az)+ip(CH) | 1350.1 | 1345.6 | 1361 m | 1349.8 | 1351.5 | 1359 m |
| rd(sa/an)+ip(CH) | 1281.3 | 1271.4 | 1273 s | 1281.4 | 1287.3 | 1283 s |
| ip(az)+ip(CH) | 1226.3 | 1228.4 | 1241 w | 1226.4 | 1226.3 | 1238 w |
| rd(sa)+ip(CH) | 1210.1 | 1214.6 | 1231 w | 1210.1 | 1213.9 | 1228 w |
| rd(sa/an)+ip(CH) | 1173.5 | 1174.2 | 1184 s | 1173.9 | 1175.2 | 1187 s |
| rd(an)+ip(CH) | 1152 | 1160.8 | 1175 s | 1153.5 | 1161.8 | 1173 s |
| rd(sa)+ip(CH) | 1138.7 | 1143.4 | 1151 m | 1138.7 | 1146.8 | 1151 m |
| rd(sa/an)+ip(CH) | 1102.4 | 1102.8 | 1120 w | 1102.4 | 1104.3 | 1105 w |
| rb(an)+str(CX) | 1064.7 | 1061.1 | 1091 s | 1047.3 | 1045.5 | 1072 s |
| rd(sa) | 1017.1 | 1019.7 | 1031 w | 1017.1 | 1019.8 | 1033 w |
| rd(an) | 991.3 | 991.5 | 1011 m | 988.0 | 990.6 | 1009 s |
| oop(az/an) | 970.3 | 969 | 963 w | 970.3 | 971 | 984 m |
| rd(sa) | 891.3 | 894.4 | 909 w | 891.2 | 891.4 | 911 m |
| oop(sa/CH/OH) |  | 852 | 845 sh. |  |  |  |
| rd(sa)+rb(an) | 836.1 | 842.2 | 838 s | 834.9 | 838.2 | 829 s |
| oop(sa/CH/OH) | 819.4 | 833.3 |  | 817.4 | 826.5 | 813 sh. |
| oop(OH) | 809.3 |  | 816 m | 807.0 | 847.9 | 849 s |
| oop(an) |  | 803.4 |  |  |  |  |
| rb(sa) | 765 | 768.2 | 780 m | 764.1 | 764.5 | 774 w |
| oop(sa) | 741.1 | 760.1 | 759 s | 741.1 | 750.2 | 753 s |
| rd(sa/an)+str(CX) | 681.9 | 682.1 | 699 s | 668.4 | 668.7 | 679 m |
| rd(sa) | 550.5 | 550.1 | 559 w | 547.5 | 551 | 552 w |
| oop(sa/an) | 521.4 | 517 | 519 m | 518.1 | 522.7 | 529 s |
| ip(CN) | 490.8 | 498.3 | 504 w | 488.7 | 494.1 | 497 w |
| ip(CO) | 438.8 | 450.7 | 447 w | 435.8 | 454.4 | 441 w |
| ip(CO)+str(CX) | 408.3 | 411.5 | 419 m | 346.4 | 350.7 |  |

str: stretching, rd: ring distortion, rb: ring breathing, ip: in-plane bending, oop: out-of-plane bending

(sa): salicylidene ring, (an): aniline ring, (az): azomethine group, (CH): aromatic C–H, (CX): C–Cl/Br, (CO): aromatic C–O, (CN): aromatic C–N

s: strong, m: medium, w: weak, sh.: shoulder

**Figure S1** α-SA4B vibration-mode correlation between isolated/in cluster molecules.

**Figure S2** β-SA4C vibration-mode correlation between isolated/in cluster molecules.

**Figure S3** The comparison of calculated IR result for α-SA4C, β-SA4C and α-SA4B based on ONIOM cluster model.

**Figure S4** PES curves of the enol- and keto-forms relevant to the pedal motion involved in the cis-trans isomerization. The energies were calculated for the high-layer (central molecule) in each cluster model of the designated crystal by B3LYP/6-311G**.


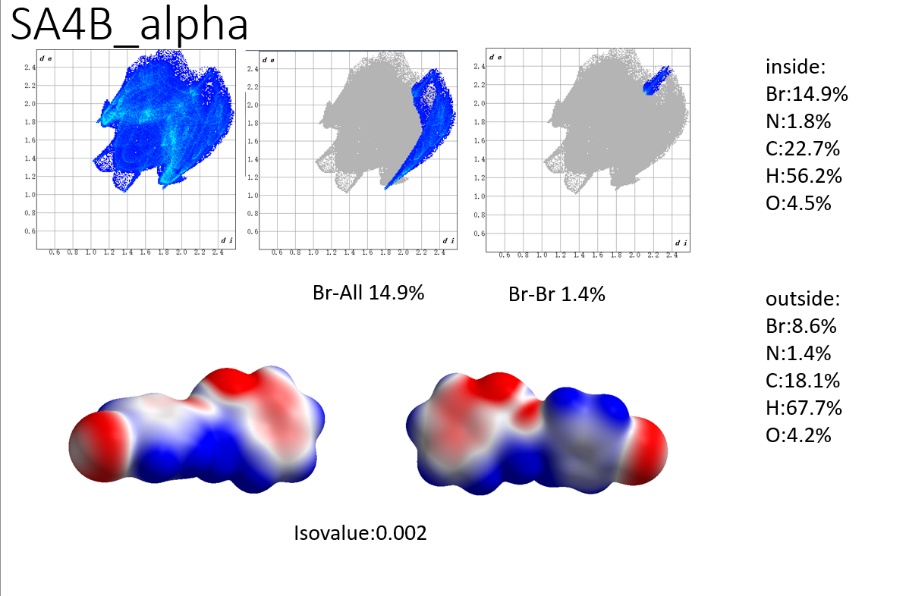


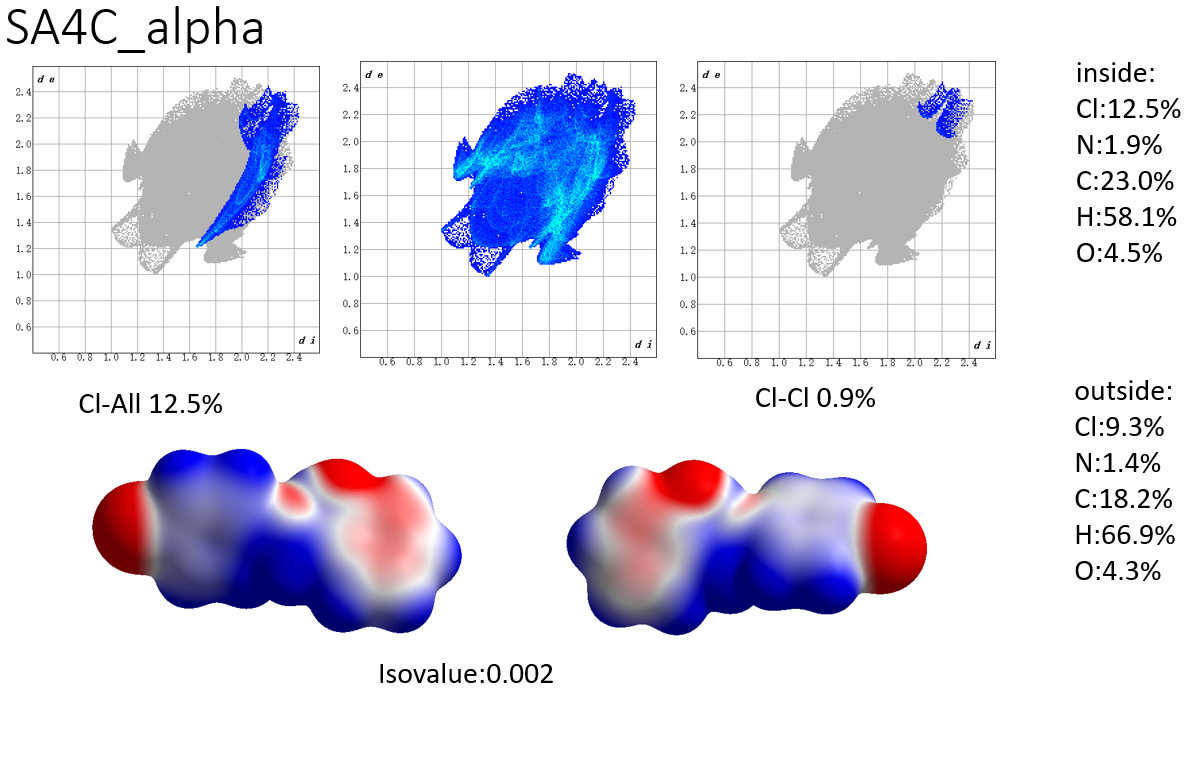


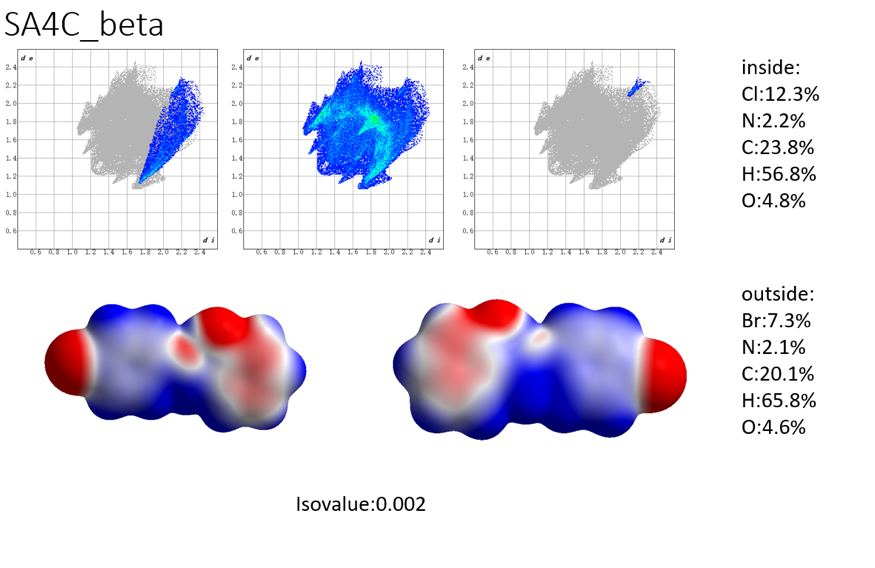


**Figure S5** Hirshfield analysis of SA4 series crystals.
